# International Clinics

**Published:** 1892-09

**Authors:** 


*International Clinics.* Vol. III., pp. vii., 372. Vol. IV., pp. xiii., 382. A Quarterly of Clinical Lectures by Professors and Lecturers in the leading Medical Colleges of the United States, Great Britain, and Canada. Edinburgh: YOUNG J. PENTLAND. 1892.

The publishers are to be congratulated on these handsome volumes, but we confess to a feeling of disappointment that so few of the lectures are from the best known workers. Such a publication might well be a permanent record of the very best clinical lectures given in the English language. The work contained in the books before us might with advantage be more international. While thirteen lectures emanate from England, no less than seventy are from the United States, and three are Canadian.

We expect original papers to contain the theories and dogmas of their authors, but clinical lectures should express what may be considered the recognised knowledge of the subject dealt with. The experiences of the laboratory and of the wards, the records of the value of therapeutics and of operations and of the pathological changes found under similar conditions must all be elaborated in order that the main points for practical diagnosis and treatment may be given in the lecture, to the best advantage of the senior students or junior practitioners for whom it is intended. The volumes before us contain many such clinical lectures of the best type. Among these may be mentioned "Tubercular Peritonitis," "Removal of the Uterine Appendages," "Hydronephrosis," "The Arm Palsies," and "Migraine."

The editors of *International Clinics* may be assured of success if they will only publish what is the very best or if they make exceptions only in the case of the best known teachers. There is still room in the midst of our numberless medical publications for the definite teachings of the clinical lecture, as the busy practitioner has little time for the study of monographs or transactions of societies, and he will readily avail himself of the knowledge of those best qualified to select and assimilate that which is likely to be useful for his practice. *International Clinics* must maintain a very high standard or they will not be read; but they may be edited so as to fulfil a real want in medical literature.

